# Clinical Assessment of Motor Function: A Processes Oriented Instrument Based on a Speed-Accuracy Trade-Off Paradigm

**DOI:** 10.1155/2007/203828

**Published:** 2007-02-12

**Authors:** Blaise Christe, Pierre R. Burkhard, Alan J. Pegna, Eugene Mayer, Claude-Alain Hauert

**Affiliations:** ^1^Neuropsychology UnitDepartment of NeurologyGeneva University Hospitals and Medical SchoolSwitzerland; ^2^Neurology Outpatients' ClinicDepartment of NeurologyGeneva University Hospitals and Medical SchoolSwitzerland; ^3^Laboratory of Experimental NeuropsychologyDepartment of NeurologyGeneva University Hospitals and Medical SchoolSwitzerland; ^4^Faculty of Psychology and Sciences of EducationUniversity of GenevaSwitzerland

**Keywords:** Motor control, clinical assessment, cognitive approach, speed-accuracy trade-off

## Abstract

In this study, we developed a digitizing tablet-based instrument for the clinical assessment of human voluntary movements targeting motor processes of planning, programming and execution. The tool was used to investigate an adaptation of Fitts' reciprocal tapping task [10], comprising four conditions, each of them modulated by three indices of difficulty related to the amplitude of movement required. Temporal, spatial and sequential constraints underlying the various conditions allowed the intricate motor processes to be dissociated. Data obtained from a group of elderly healthy subjects (*N* = 50) were in agreement with the literature on motor control, in the temporal and spatial domains. Speed constraints generated gains in the temporal domain and costs in the spatial one, while spatial constraints generated gain in the spatial domain and costs in the temporal one; finally, sequential constraints revealed the integrative nature of the cognitive operations involved in motor production. This versatile instrument proved capable of providing quantitative, accurate and sensitive measures of the various processes sustaining voluntary movement in healthy subjects. Altogether, analyses performed in this study generated a theoretical framework and reference data which could be used in the future for the clinical assessment of patients with various movement disorders, in particular Parkinson’s disease.

## 1. Introduction

The clinical assessment of movement disorders is currently based on qualitative and subjective evaluation of muscle control, strength and tone, obtained in routine neurological examinations, and on single ordinal tests or scales focused on the final steps of movement processing (task-oriented). On the other hand, pre-execution, cognitive steps (process-oriented) remain virtually ignored. In addition, the range of movements is so diversified that no task-oriented test can cover it exhaustively [15,35], and only a comprehensive analysis of all motor processes underlying any overt motor response is likely to capture selectively the various pathologies seen in the clinic and to assess global motor function. For this purpose, we developed a new instrument based on a cognitive approach of motor control. This instrument was designed to provide quantified information related to distinct motor processes, to be flexible in its breadth of application, and to comply with the financial and time demands associated with clinical practice.

ISSN 0953-4180/07/$17.00 © 2007 – IOS Press and the authors. All rights reserved

## 2. Background

### 2.1. Motor control

Although many issues remain debated in the field of motor control, serial stage models have emerged over the last quarter of century (see, for example [24–26,33, 34]) constituting a heuristic framework which allows a simple conceptualization of the principal stages of information processing, leading ultimately to the production of voluntary movements. According to these models, voluntary movements appear to originate from an exogenous or endogenous stimulus that, once detected and identified, becomes a mandatory signal. In a subsequent planning stage, subjects select the best abstract response plan leading to the targeted goal associated to the mandatory signal. During the programming stage, this abstract response plan is implemented into a motor code where the temporal and spatial parameters of movement segments are determined. Figuratively speaking, planning corresponds to the strategic stage and programming to the tactical stage. In the execution stage, the motor code becomes an overt response through activation of adequate muscular effectors. Finally, the feedback stage, which includes detection and error correction, allows a constant fine-tuning of the ongoing movement. This conception of motor control brings forward the idea of a central organization of movements (open-loop models), integrating sensory information in movement regulation (closed-loop models). This cognitive concept of motor control is in agreement with the current knowledge of the general anatomy and physiology of the human motor system (see, for example [2,4,7,20,21]).

### 2.2. Speed-accuracy trade-off problem

The speed-accuracy trade-off offers a useful framework in which to study movement processes at the clinical level. It reflects the everyday compromise in human behavior, as indeed conflicting constraints of speed and spatial accuracy require constant modulation of voluntary movement (see, for example [17,18]). In fact, due to signal-dependent noise in the neural control signal, the reduction of movement time in a given motor task increases spatial variability. Conversely, the reduction of spatial variability increases movement time (see, for example [13,17–19]).

An additional advantage for the study of movement resides in the precision of mathematical formalizations and the consistency of empirical results related to the speed-accuracy trade-off. Along these lines, Fitts [9] established formulae that allowed (i) the quantification of an Index of Difficulty (ID) of a motor task as a function of amplitude (A) and tolerance (W, i.e., the required spatial accuracy) of movement (Eq. (1)), (ii) the definition of the evolution of movement time (MT) in relation to this difficulty (Eq. (2)) and (iii) the quantification of the information processing capacity (C) of the subject under study (Eq. (3)).

$$ID = \log_2 \frac{2 \times A}{W} \text{ [bits]} \quad (1)$$

$$MT = a + b \times ID \text{ [s]} \quad (2)$$

$$C = \frac{ID}{MT} \, [bits/s] \quad (3)$$

## 3. Current research

In an attempt to overcome the current limitations of motor assessment in clinical practice, we applied Fitts' reciprocal tapping task [9] on a digitizing tablet and adapted it so as to focus on specific aspects of motor processing, namely planning, programming and execution.

As detailed below in the Method section, the involvement of each of these motor processes is dependent on the temporal, spatial and sequential constraints included in four distinct experimental conditions. The "execution" condition was characterized by weak spatio-temporal constraints, as well as the absence of a strategy-based response with respect to target choice. In the "speed programming" condition, we included a temporal constraint (time minimization). In the "speed-accuracy programming" condition, additional spatial constraints were incorporated (objective spatial accuracy). Finally, in the "planning" condition, sequences of different movement segments were required, entailing a strategy-based response for target choice.

We expected that the differential implication of planning, programming and execution processes allowed us, on the one hand, to confirm that speed constraints generate gains in the temporal domain and, on the other hand, that spatial constraints produce a gain in the spatial field but a cost in the temporal one. Finally, we expected to establish, via the presence or absence of a context-dependent evolution of movement time [23], the role of cognitive planning operations in complex sequential motor behaviors.

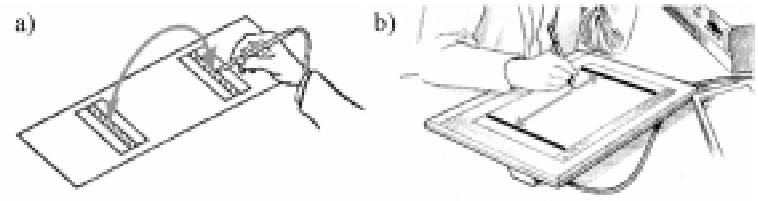

Fig. 1. Representation a) of Fitts reciprocal tapping task [10] and b) of a typical situation using a digitizing tablet. The double arrows indicate the periodic movements between the two targets (hatched or black bands).

We evaluated elderly participants because they represent the most likely age sample for common movement disorders associated with neurological diseases, notably Parkinson's disease. Consequently, we investigated the potential effect of the demographic factors of gender and age on motor performance.

## 4. Method

### 4.1. Assessment tool

#### 4.1.1. Application of Fitts reciprocal tapping task on a digitizing tablet

The Fitts' reciprocal tapping task [9] consists in tapping a stylus alternately between two targets (Fig. 1a). It is a spatially constrained paradigm where the participants have to minimize the average duration of their movements while preserving a high level of spatial precision. Equation (1) allows a definition of task difficulty using tolerance (width of the targets) and amplitude (distance separating the centre of the targets) of movement.

We applied this paradigm on a digitizing tablet by replacing the two target plates by black bands printed on a sheet of paper placed on its sensitive surface (Fig. 1b). Applied in this manner, the paradigm preserved its cardinal features. We used the possibility of controlling task difficulty by manipulating the amplitude of movement, and/or the tolerance of the targets (Eq. (1)) to create three distinct indices of difficulty (Fig. 2). For practical reasons discussed below, task difficulty was modulated by varying movement amplitude ($A = 56.5$ mm, 113.0 mm and 226.0 mm), while tolerance of the targets was kept constant ($W = 10.0$ mm). Indices of difficulty of 3.5, 4.5 and 5.5 bits allowed us to target the different operating modes related to periodic movements, described by Guiard [11,12]: the harmonic, transient and inharmonic modes. These modes are defined respectively by a maximum, intermediate and minimal recuperation rate of the deceleration energy of a given movement for the acceleration in the subsequent opposite movement. Furthermore, the periodic nature of the movements allowed data collection to be optimized in relation to the number of trials carried out. This is an important parameter in clinical practice where the difficulties in understanding and initiating the tasks required are relatively common in certain patients. Finally, as the aiming movements were performed with the stylus in constant contact with the surface of the digitizing tablet, disturbing impact movements were excluded (see, for example [10,37]).

#### 4.1.2. Adaptation of Fitts' reciprocal tapping task to assess motor processes

In order to evaluate the motor processes of planning, programming and execution separately, four experimental conditions were designed (Fig. 2). The transposition of Fitts' reciprocal tapping task on the digitizing tablet constituted the speed-accuracy programming condition (Fig. 1b). As participants had to minimize the average duration of movement while maintaining the highest degree of spatial accuracy, the constraints in this condition were related to the implementation of both the temporal and spatial motor program parameters. With the exception of execution processes, which are present in all overt motor behavior, the choice of a single target restricted all answering strategies and consequently all planning processes.

In comparison with the speed-accuracy programming condition, the speed programming and execution conditions were characterized by a reduction in programming processes. In accordance with Sheridan, Flowers and Hurrell [30], this reduction was carried out by minimizing movement parameters constraints. In the speed programming condition, the constraints in spatial accuracy were reduced by replacing the black bands representing the targets, by two strips that showed a graded black-to-white border on both sides. With these graded stimuli, participants were asked to point within what they perceived as the darkest area. It should be noted that the median width of this

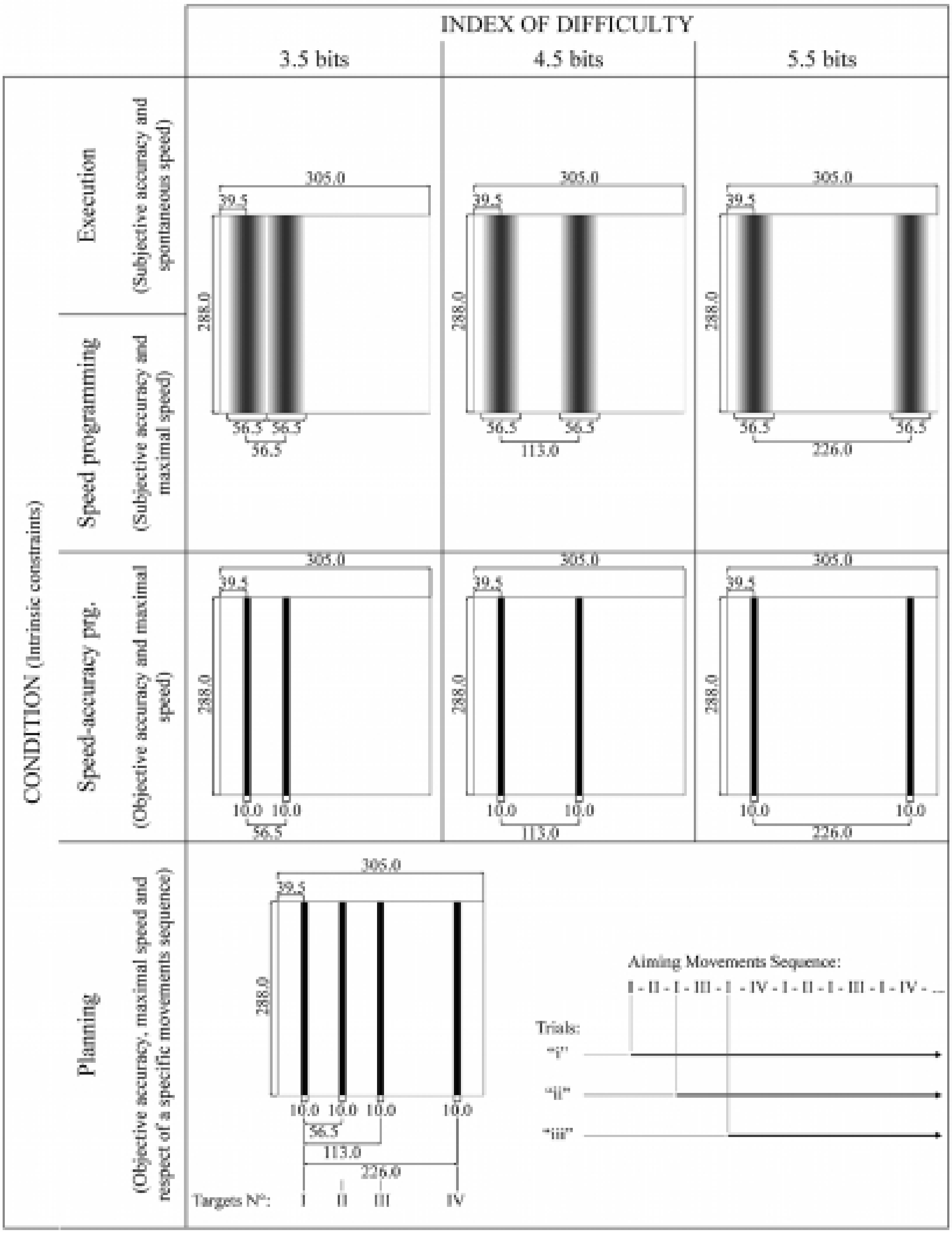

Fig. 2. Representation of the target sheets (dimensions in mm) which, depending on their pattern and the instructions given, determine four experimental conditions and three indices of difficulty. Planning condition: diagram of the sequence course according to the experimental trial.

darkest area was estimated with a ruler at 11 mm by 10 healthy participants, i.e., similar to the objective width of the black bands.

In addition to this reduction of spatial constraints, the execution condition included a reduction of temporal constraints as well, as the participants no longer had to minimize pointing duration but were asked to adopt a spontaneous, comfortable speed. In the absence of a strategy for response choice and of strong constraints concerning the temporal and spatial implementation of the motor programs, the label "execution" was chosen to emphasize the process of translation of the motor code into an overt motor response.

Compared to the speed-accuracy programming condition, the planning condition was further complicated by increasing the number of response options. In practice,the response sheet in the planning condition included all the targets of the speed-accuracy programming condition. The left-hand targets in the speed-accuracy programming condition being placed at the same location, the sheet in the planning condition comprised only four targets to which the participants were required to point in a predefined sequence. This motor sequence was comparable to the aiming movements in the speed-accuracy condition alternating in ascending order of difficulty, i.e., the sequence of movements "3.5 bits – 4.5 bits – 5.5 bits. . . " (Fig. 2).

### 4.2. Procedure

The digitizing tablet (WACOM UltraPad A4) was set on a desk in front of the participant, positioned in such a manner that the motor productions were performed in the axis of minimal inertia.

A complete evaluation required 12 trials, the participants being evaluated in four conditions combined with three indices of difficulty. Although each single trial included all three indices in the planning condition, three trials were carried out in order to have the same temporal allocation per target as the other conditions. All three trials in this condition related to the same aiming sequence (3.5 bits – 4.5 bits – 5.5 bits . . . ), but only the initial movement differed: respectively, 3.5 bits, 4.5 bits or 5.5 bits (Fig. 2). Assessment was made by ascending order of complexity of the processes (execution, speed programming, speed-accuracy programming and planning) and by ascending order of difficulty of movement. Although this induced a training bias, the absence of order randomization was considered as the only solution allowing comparisons of single case results with a control population.

Each trial lasted 10 s and was preceded by a familiarization trial of equivalent duration. The data acquisition was automatically synchronized with the occurrence of an auditory "go" signal.

### 4.3. Data processing

For each trial, the spatial coordinates of the stylus were recorded with an acquisition frequency of 200 Hz and a spatial resolution of 0.25 mm. Only the data concerning the main axis of movement (the x-axis) were taken into account. The signal of the digitizing tablet was filtered to obtain data concerning position. We used a 199-point long low-pass filter of the "Finite Impulse Response" type, without phase displacement. The band pass was set from 0 to 10 Hz, its minimum answer gain was $-0.09$ dB. The transition band ranged from 10 to 12 Hz. Finally, the maximum attenuation gain of the stop band was $-40.00$ dB.

### 4.4. Research participants

Performances of a group of 50 elderly healthy subjects (mean age $=$ 68.0 years; $sd = 7.4$ years; sex ratio $=$ 1) were evaluated. Ninety-eight percent were right-handed as defined by the Bryden questionnaire [5]. All denied any past or present neurological, rheumatic or surgical condition that may have influenced motor function. In order to investigate the influence of Gender and Age, this sample was divided according to age into two groups of 25 individuals each. The group of younger subjects ($m = 62.5$ years; $sd = 1.4$ years) was composed of 60% males, 98% right-handed and 2% left-handed. The group of older subjects ($m = 73.5$ years; $sd = 6.4$ years) was composed of 40% males, and 100% right-handed.

This experiment was approved by the Ethics Committee of the Department of Clinical Neurosciences of the Geneva University Hospitals as well as by the Ethics Committee of the Faculty of Psychology and Educational Sciences of the University of Geneva.

### 4.5. Measures

Results of the productions of the participants' dominant hand are presented here in the form of three measures describing the motor performances from the temporal (movement time) and spatial (constant error, variable error) domains:

- movement time is defined as the mean average duration of the half-cycles of movement (from one spatial extreme to the next) per participant;
- constant error refers to the mean distance by subject between movement endpoints and target center. Overshoot errors are indicated by positive values and undershoot errors by negative values;
- variable error is the standard deviation of the subject's own mean movement endpoints.

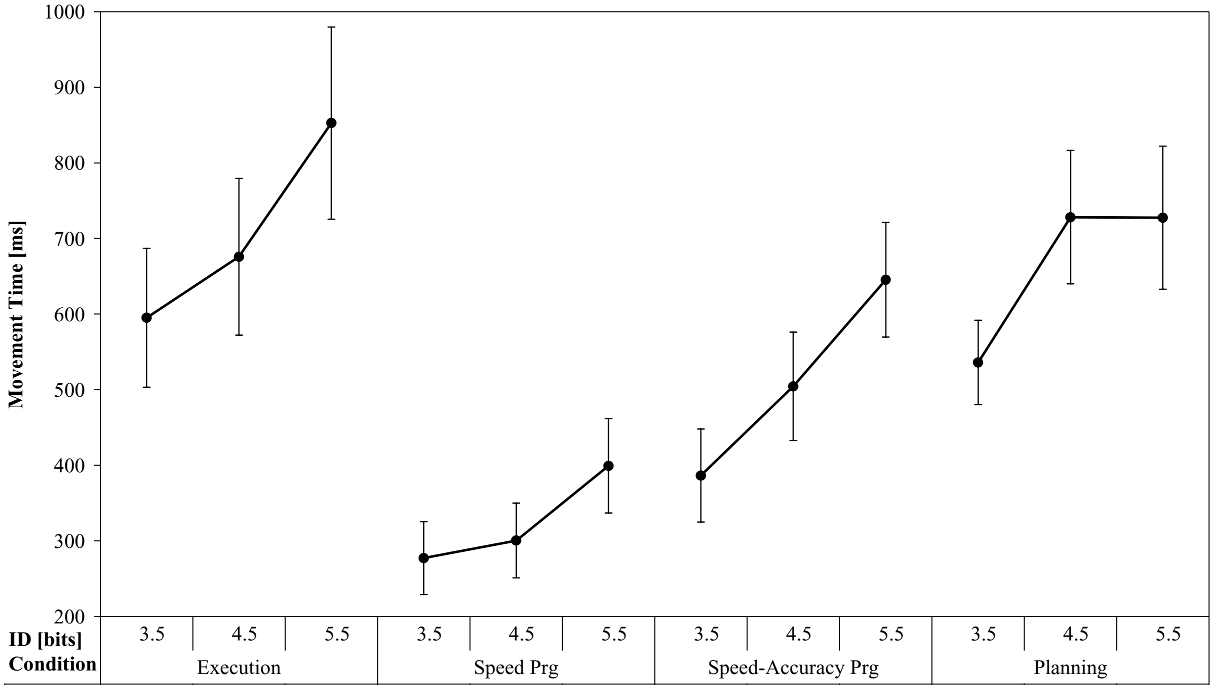

| Condition | Execution | | | Speed Prg | | | Speed-Accuracy Prg | | | Planning | | |
|---|---|---|---|---|---|---|---|---|---|---|---|---|
| ID [bits] | 3.5 | 4.5 | 5.5 | 3.5 | 4.5 | 5.5 | 3.5 | 4.5 | 5.5 | 3.5 | 4.5 | 5.5 |
| Mean | 595 | 676 | 853 | 277 | 300 | 399 | 386 | 504 | 645 | 536 | 728 | 727 |
| Sd | 244 | 275 | 338 | 128 | 131 | 166 | 163 | 191 | 202 | 149 | 234 | 251 |

Fig. 3. Evolution of movement time as a function of Condition and Index of Difficulty (ID) / Graphic: mean and 99% confidence interval for mean (error bars) / Table: mean and standard deviation (Sd).

*4.6. Statistical analyses*

To correct for potential outliers, all the residuals of the within-subjects analysis of variance (ANOVA) carried out on the factors Condition and Index of Difficulty were identified, whose standardized values were greater than 3 standard deviations. When several extreme residuals were related to the same participant, the subject was excluded from the group for the given measure. For the other extreme residuals, the original scores were corrected using the extrapolation of the Eq. (4).

$$\mathrm{Score}_{\mathrm{Subject}_n \mathrm{Cond}_j \mathrm{ID}_{x'}} \rightarrow \mathrm{Mean}_{\mathrm{Cond}_j \mathrm{ID}_{x'}} \times \left( \frac{\mathrm{Score}_{\mathrm{Subject}_n \mathrm{Cond}_j \mathrm{ID}_{x''}} + \mathrm{Score}_{\mathrm{Subject}_n \mathrm{Cond}_j \mathrm{ID}_{x'''}}}{\mathrm{Mean}_{\mathrm{Cond}_j \mathrm{ID}_{x''}} + \mathrm{Mean}_{\mathrm{Cond}_j \mathrm{ID}_{x'''}}} \right) \quad (4)$$

In this equation n is the number of the participant obtaining an extreme value in the condition $j$ for a difficulty $x'$; $x''$ and $x'''$ indicate the two other difficulties in the same condition for which this participant had acceptable scores.

The alpha level for this research was fixed at 0.01. This level was corrected using the Bonferroni's method for multiple and non-orthogonal a priori comparisons. When the hypotheses included all possible pairwise comparisons we opted for the Tukey's Honestly Significant Difference (HSD) test to reduce the family-wise type I error. Finally, we adopted the Greenhouse-Geisser correction for the ANOVA in which the sphericity assumption was not assumed.

## 5. Results

*5.1. Movement time*

The within-subjects ANOVA on movement time was significant for the main effects of Condition $[F(3, 138) = 63.72$, epsilon $= 0.61$, $p < 0.001]$ and Index of Difficulty $[F(2, 92) = 145.76$, epsilon $= 0.80$, $p < 0.001]$ factors, as well as for their interaction $[F(6, 276) = 16.35$, epsilon $= 0.67$, $p < 0.001]$. Figure 3 illustrates the evolution of movement time with Condition and Index of Difficulty.

For the factor Condition, unilateral multiple comparisons (Tukey's HSD test) revealed a significant increase in movement time between speed programming,

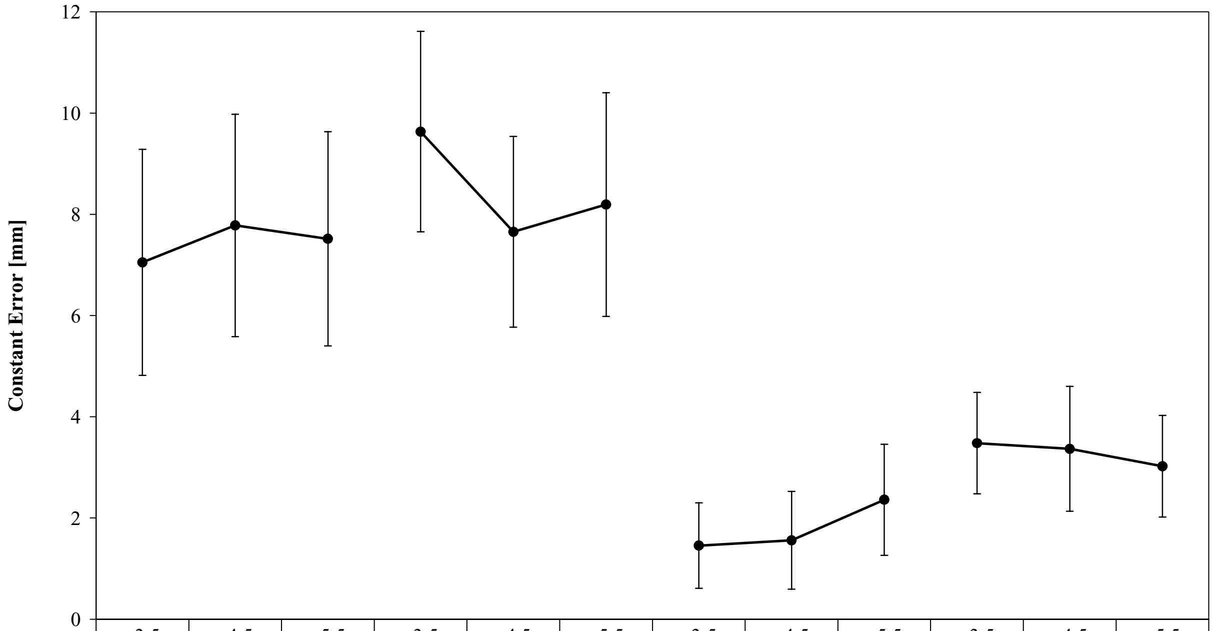

| ID [bits] | 3.5 | 4.5 | 5.5 | 3.5 | 4.5 | 5.5 | 3.5 | 4.5 | 5.5 | 3.5 | 4.5 | 5.5 |
|---|---|---|---|---|---|---|---|---|---|---|---|---|
| Condition | Execution | | | Speed Prg | | | Speed-Accuracy Prg | | | Planning | | |
| Mean | 7.0 | 7.8 | 7.5 | 9.6 | 7.7 | 8.2 | 1.5 | 1.6 | 2.4 | 3.5 | 3.4 | 3.0 |
| Sd | 6.0 | 5.9 | 5.7 | 5.3 | 5.1 | 5.9 | 2.3 | 2.6 | 2.9 | 2.7 | 3.3 | 2.7 |

Fig. 4. Evolution of constant error as a function of Condition and Index of Difficulty (ID) / Graphic: mean and 99% confidence interval for mean (error bars) / Table: mean and standard deviation (Sd).

speed-accuracy programming and planning conditions ($p < 0.001$ for all comparisons). Although movement time in the execution condition is higher than that of the planning condition, this difference is not significant ($p = 0.238$).

For the factor Index of Difficulty, unilateral multiple comparisons (Tukey's HSD test) demonstrated an increase in movement time through 3.5 bits, 4.5 bits and 5.5 bits ($p <$ 0.001 for all comparisons).

Regarding the Condition x Index of Difficulty interaction, a contrasts analysis revealed a smaller increase of movement time in the planning compared to the speed-accuracy conditions for the highest Index of Difficulty, when comparing with the two other indices taken together $[F(1, 46) = 15.54,\ p < 0.001]$.

To assess whether these results were compatible with Fitts' law, the linearity of the evolution of movement time for the speed-accuracy programming condition was tested by comparing the average movement time of the two extreme difficulties taken together, with the average movement time for the intermediate difficulty. The absence of significance in a bilateral $t$ test $[t(46) = -1.38,\ p = 0.175]$ suggests a linear trend in task difficulty with the evolution of movement time.

### *5.2. Constant error*

The within-subjects ANOVA concerning constant error showed only a significant main effect for Condition $[F(3, 141) = 44.45$, epsilon $= 0.69$, $p < 0.001]$. Index of Difficulty, as a main effect $[F(2, 94) = 0.91,\ p = 406]$, and the interaction Condition x Index of Difficulty effect $[F(6, 282) = 3.48$, epsilon $= 0.59$, $p = 0.012]$ were not significant. Figure 4 illustrates the evolution of constant error as a function of Condition and Index of Difficulty.

Multiple comparisons (Tukey's HSD test) did not show any significant advantage of spatial accuracy in the execution compared to the speed programming conditions ($p_{\text{unilat.}} =$ 0.210). Spatial accuracy was systematically higher for speed-accuracy programming and planning conditions than for execution and speed programming conditions ($p_{\text{unilat.}} <$ 0.001 for both comparisons). The constant error in the planning condition did not differ significantly from that of the speed-accuracy programming condition ($p_{\text{bilat.}} =$ 0.123).

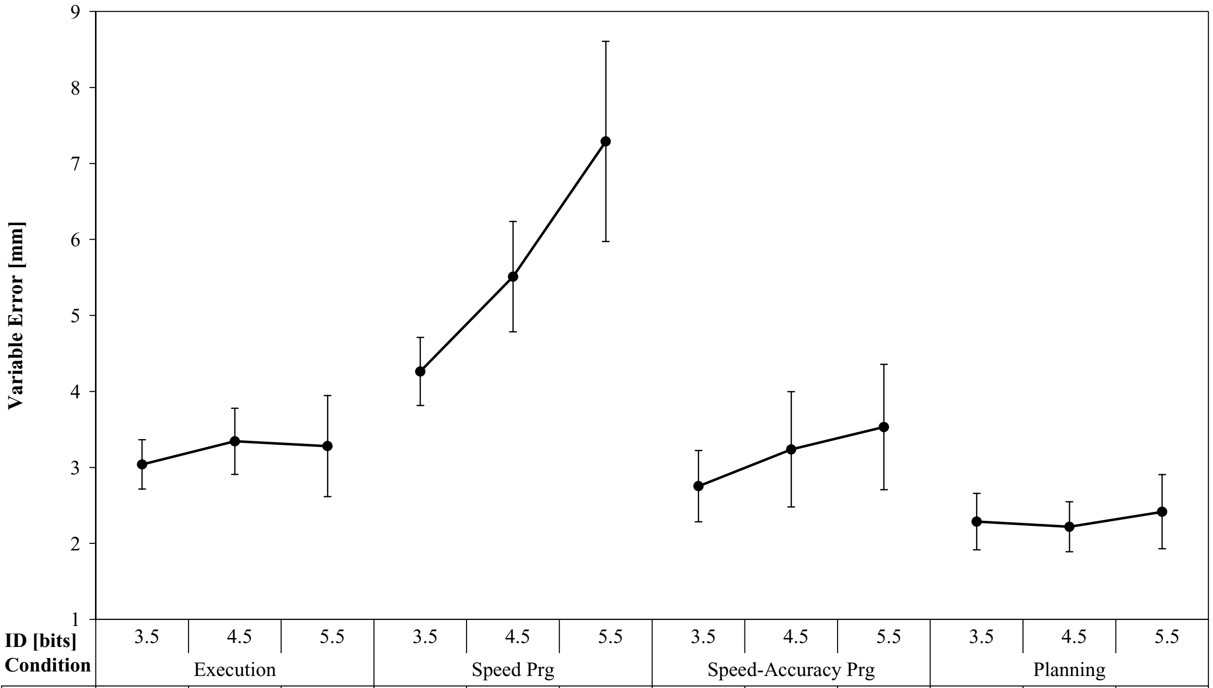

| | Execution 3.5 | Execution 4.5 | Execution 5.5 | Speed Prg 3.5 | Speed Prg 4.5 | Speed Prg 5.5 | Speed-Accuracy Prg 3.5 | Speed-Accuracy Prg 4.5 | Speed-Accuracy Prg 5.5 | Planning 3.5 | Planning 4.5 | Planning 5.5 |
|---|---|---|---|---|---|---|---|---|---|---|---|---|
| Mean | 3.0 | 3.3 | 3.3 | 4.3 | 5.5 | 7.3 | 2.8 | 3.2 | 3.5 | 2.3 | 2.2 | 2.4 |
| Sd | 0.9 | 1.2 | 1.8 | 1.2 | 2.0 | 3.6 | 1.3 | 2.1 | 2.2 | 1.0 | 0.9 | 1.3 |

Fig. 5. Evolution of variable error as a function of Condition and Index of Difficulty (ID) / Graphic: mean and 99% confidence interval for mean (error bars) / Table: mean and standard deviation (Sd).

### 5.3. *Variable error*

The within-subjects ANOVA demonstrated significant main effects of Condition $[F(3,120) = 80.45$, epsilon $= 0.79$, $p < 0.001]$ and Index of Difficulty $[F(2,80) = 22.24$, epsilon $= 0.74, p < 0.001]$, as well as a significant Condition x Index of Difficulty interaction $[F(6,240) = 14.73$, epsilon $= 0.64$, $p < 0.001]$. Figure 5 illustrates the evolution of variable error as a function of Condition and Index of Difficulty.

Regarding the factor Condition, unilateral multiple comparisons (Tukey's HSD test) showed a maximal spatial variability for the speed programming condition ($p < 0.001$ for all comparisons including this condition). The remaining results suggested a smaller variable error for the planning with regard to the execution ($p_{\mathrm{bilat.}} = 0.001$) and the speed-accuracy programming ($p_{\mathrm{bilat.}} < 0.001$) conditions, these last two showing no significant difference between them ($p_{\mathrm{bilat.}} = 0.982$).

In relation to the Index of Difficulty, unilateral multiple comparisons (Tukey's HSD test) demonstrated an increase of spatial variability with amplitude of movement (3.5 bits vs 4.5 bits, $p = 0.003$ / 4.5 bits vs 5.5bits, $p = 0.001$ / 3.5 bits vs 5.5 bits, $p < 0.001$).

Finally, with respect to the two factors interaction, a priori multiple contrasts analyses (alpha level next Bonferroni correction = 0.003) revealed a stronger increase of the variable error with the Index of Difficulty in the speed programming as compared to the other conditions ($p < 0.001$ for all contrasts).

### 5.4. *Gender and age*

A four-way mixed-design ANOVA, with Age and Gender as between-subject factors and Condition and Index of Difficulty as within-subject factors, showed the same main effects and interactions as the principal statistical analyses described above. This was true for the three measures observed: movement time, constant and variable errors. Concerning the between-subject factors, this analysis only revealed the main effect of Age on movement time $[F(1,43) = 13.50$, $p = 0.001]$, with a lower mean movement time for the younger group ($m = 485.78$ ms; $sd = 27.65$ ms) in comparison to the older one ($m = 633.03$ ms; $sd = 29.00$ ms). No mixed interaction was observed. Table 1 reports descriptive results of movement time in relation to Condition, Index of Difficulty and Age factors.

Table 1
Descriptive statistics concerning factors Condition, Index of Difficulty (ID) and Age for movement time (Sd = standard deviation; C. I. = confidence interval)

| Age group [years] | Condition | ID [bits] | N | MOVEMENT TIME [ms] Mean | Sd | C. I. for mean (1%) | (99%) |
|---|---|---|---|---|---|---|---|
| Mean = 62.5 | Execution | 3.5 | 25 | 515.52 | 198.99 | 404.21 | 626.83 |
| (Min = 60.2, | | 4.5 | 25 | 585.37 | 224.77 | 459.64 | 711.11 |
| Max = 65.1) | | 5.5 | 25 | 723.65 | 277.33 | 568.52 | 878.79 |
| | Speed Prg | 3.5 | 25 | 245.87 | 110.58 | 184.01 | 307.72 |
| | | 4.5 | 25 | 267.75 | 114.30 | 203.81 | 331.69 |
| | | 5.5 | 25 | 359.34 | 148.30 | 276.39 | 442.30 |
| | Speed-Accuracy Prg | 3.5 | 25 | 324.35 | 148.97 | 241.02 | 407.68 |
| | | 4.5 | 25 | 407.63 | 156.67 | 319.99 | 495.27 |
| | | 5.5 | 25 | 551.33 | 178.50 | 451.48 | 651.18 |
| | Planning | 3.5 | 25 | 489.18 | 149.73 | 405.42 | 572.94 |
| | | 4.5 | 25 | 653.28 | 180.72 | 552.19 | 754.37 |
| | | 5.5 | 25 | 636.97 | 193.39 | 528.79 | 745.15 |
| Mean = 73.5 | Execution | 3.5 | 22 | 685.33 | 263.43 | 526.30 | 844.35 |
| (Min = 65.8, | | 4.5 | 22 | 778.35 | 296.22 | 599.54 | 957.16 |
| Max = 85.8) | | 5.5 | 22 | 999.29 | 346.02 | 790.42 | 1208.1 |
| | Speed Prg | 3.5 | 22 | 312.48 | 139.43 | 228.31 | 396.65 |
| | | 4.5 | 22 | 337.31 | 142.25 | 251.44 | 423.18 |
| | | 5.5 | 22 | 444.27 | 176.15 | 337.94 | 550.60 |
| | Speed-Accuracy Prg | 3.5 | 22 | 456.60 | 153.07 | 364.20 | 549.00 |
| | | 4.5 | 22 | 614.30 | 166.86 | 513.57 | 715.03 |
| | | 5.5 | 22 | 752.24 | 173.36 | 647.59 | 856.89 |
| | Planning | 3.5 | 22 | 589.20 | 131.21 | 509.99 | 668.40 |
| | | 4.5 | 22 | 813.01 | 262.08 | 654.81 | 971.22 |
| | | 5.5 | 22 | 830.16 | 273.88 | 664.83 | 995.49 |

## 6. Discussion

The factors Condition and Index of Difficulty that aimed respectively at differentiating the various motor processes involved in the task, and at modulating intrinsic task complexity, yielded results that can be interpreted temporally and spatially.

### 6.1. Movement time

The contrasts analysis on the Condition x Index of Difficulty interaction showed, for a motor sequence of varying difficulty, that the movement time of one segment is influenced by the features of other segments. This result indicates the presence of a "context-dependent effect" [1,16,23] related to the planning condition. According to Rand et al. [23], such an effect establishes that the motor planning process starts prior to the initiation of the motor sequence, and considers the features of all movement segments in order to determine the strategy of the entire sequence.

With one exception, statistical analyses taking into account all indices of difficulty confirmed the expected influences of experimental constraints regarding the effect of Condition. Movement times were shortest for the speed programming condition; they increased in the speed-accuracy condition and even more so in the planning condition. De facto, movement time decreased if speed of movement was emphasized, but increased if higher spatial accuracy or higher sequence complexity was required. The single exception concerned movement times in the execution condition for which we had hypothesized an increase due to the absence of any temporal constraint. Indeed, from the temporal viewpoint, it appears that the management of sequential constraints is as costly as the absence of temporal constraints.

Statistical analyses supported the assumption of an increase in movement time with greater Index of Difficulty. In the speed-accuracy condition, results corroborated the linear evolution of movement time with task difficulty as formalized by Fitts' law (Eq. (2)).

### 6.2. Constant error

The only effect anticipated was related to Condition, as the presence of spatial constraints was expected to minimize the spatial bias, and the presence of temporal constraints to increase it. Although Schmidt and collaborators showed an increase in neuromuscular noise with greater amplitudes of movement [28,29], we did not expect any significant systematic spatial bias due to the Index of Difficulty because of the stochastic nature of this neuromuscular noise. Due to the fact that, in the planning condition, target tolerance (width) was identical, we did not expect a Condition x Index of Difficulty interaction effect [14,31].

Our results confirmed a global effect of Condition, but multiple comparisons did not entirely support the initial hypotheses. Indeed, although we observed the expected systematic effect of spatial constraints on constant error, we also observed an unexpected preservation of spatial accuracy when temporal constraints were added. The absence of an increase in constant error between the execution and speed conditions can be explained as a speed-accuracy trade-off problem. The absence of both objective spatial constraints and temporal ones appears to preserve the execution condition from a speed-accuracy conflict because this condition does not force the motor system to its limit. This lack of saturation enables the motor system to heighten movement speed without downgrading spatial accuracy.

### *6.3. Variable error*

The statistical analyses confirmed the effect of Condition on spatial variability and, more specifically, the fact that the latter is maximal in the speed programming condition. As expected (see, for example [17,18, 28,29,32]), the reduced spatial constraints in association with constraints of temporal minimization induced a more important variable error than in the other conditions. The opposing effects of spatial and temporal constraints on spatial variability could also explain the absence of any significant difference between execution and speed-accuracy programming conditions. Along the same lines, we observed an unexpected difference between the speed-accuracy and planning conditions, with a smaller variable error for the latter.

Statistical analyses supported the assumption of an increase in variable error with greater Indices of Difficulty, an evolution explained by amplification of noise in the neural control signal with increasing amplitude of movement [28,29]. Finally, the contrasts analyses confirmed the interaction of the Condition and Index of Difficulty effects, with the strongest increase in spatial variability with increasing amplitude of movement observed in the speed programming condition.

### *6.4. Demographical considerations*

Secondary investigations regarding the demographical influences of Gender and Age factors revealed only a main effect of Age on movement time. Considering the rather controversial dataset on a potential effect of Gender on motor performances (see, for example [27]), the absence of a significant difference related to this factor was not surprising. With respect to the Age factor on the other hand, an increased movement time with age appears to be consistent with the generally accepted view of a global slowing of voluntary movement in the elderly. It is therefore possible, given a larger sample size and a broader age range, that stronger and more specific effects might appear in relation to sensorimotor, cognitive and strategy changes known to occur with age (see, for example [36,38]).

## 7. Conclusion

The results obtained in this study – especially those concerning the differential involvement of planning, programming and execution processes included in the Condition factor – constitute an original set of reference data to which the motor performance of patients with various movement disorders of neurological origin can be compared, including pyramidal, extrapyramidal and cerebellar dysfunctions. Practically, based on our results, we anticipated that patients with pure high-level process disorders (planning and, to a lesser extent, programming) would show deficits in the most demanding experimental conditions (planning and, to a lesser extent, speed-accuracy programming), with a relative preservation in the other conditions. At the other end of the spectrum, the patients with low-level process disorders should show a systematic impairment with no dissociation between the various experimental conditions. To verify these assumptions, further research is currently underway in patients with Parkinson's disease in order to obtain quantitative and differential information on the akinetic-rigid syndrome and more specifically to investigate the temporal and spatial consequences of programming disruption and the sequential consequences of planning impairment [6]. In addition, we plan to use this cognitive oriented assessment tool in pathologies which are only occasionally associated with motor dysfunction, as is the case in Alzheimer's disease which, in addition to the various well-known cognitive disorders (i.e., amnesia, aphasia, agnosia or apraxia), may also show some degree of parkinsonism [22]. Indeed, motor symptoms can appear at a moderate [8], or even mild [3] stage of dementia and their estimated prevalence may only depend on the sensitivity of the tools used to detect them [8]. The instrument described here may prove effective in overcoming these limitations.

## References

[1] J.J. Adam, F.G.W.C. Paas, I.C.J.M. Eyssen, H. Slingerland, H. Bekkering and M. Drost, The control of two-element, reciprocal aiming movements: Evidence for chunking, *Hum Mov Sci* **14** (1995), 1–11.

[2] G.I. Allen and N. Tsukahara, Cerebrocerebellar communication systems, *Physiol Rev* **54** (1974), 957–1006.

[3] T. Benke, Two forms of apraxia in Alzheimer's disease, *Cortex* **29** (1993), 715–725.

[4] V.B. Brooks, *The neural basis of motor control*, Oxford University Press: New York, 1986.

[5] M.P. Bryden, Measuring handedness with questionnaires, *Neuropsychologia* **15** (1977), 617–624.

[6] B. Christe, C.-A. Hauert, A.J. Pegna, E. Mayer and P.R. Burkhard, Clinical Assessment of Motor Function: An Emphasis on Information Processing in Parkinson's Disease Akinetic Symptoms, (in preparation).

[7] M.R. DeLong, A.P. Georgopoulos and M.D. Crutcher, Cortico-basal ganglia relations and coding of motor performance, in: *Neural Coding of Motor Performance, Vol. 7, Exp Brain Res Suppl*, J. Massion, J. Paillard, W. Schultz and M. Wiesendanger, eds, Springer-Verlag: Berlin, 1983, pp. 30–40.

[8] R.J. Ellis, M. Caligiuri, D. Galasko and L.J. Thal, Extrapyramidal motor signs in clinically diagnosed Alzheimer disease, *Alzheimer Dis Assoc Disord* **10** (1996), 103–114.

[9] P.M. Fitts, The information capacity of the human motor system in controlling the amplitude of movement, *J Exp Psychol Gen* **47** (1954), 381–391.

[10] G.L. Gottlieb, Influence of strategy on muscle activity during impact movements, *J Mot Behav* **33** (2001), 235–242.

[11] Y. Guiard, On Fitts's and Hooke's laws: simple harmonic movement in upper-limb cyclical aiming, *Acta Psychol* (*Amst*) **82** (1993), 139–159.

[12] Y. Guiard, Fitts' law in the discrete vs cyclical paradigm, *Hum Mov Sci* **16** (1997), 97–131.

[13] C.M. Harris and D.M. Wolpert, Signal-dependent noise determines motor planning, *Nature* **394** (1998), 780–784.

[14] J.M. Lajoie and I.M. Franks, Response programming as a function of accuracy and complexity: Evidence from latency and kinematic measures, *Hum Mov Sci* **16** (1997), 485–505.

[15] S.D. MacPhee, Functional hand evaluations: a review, *Am J Occup Ther* **41** (1987), 158–163.

[16] R.G. Marteniuk, C.L. MacKenzie, M. Jeannerod, S. Athenes and C. Dugas, Constraints on human arm movement trajectories, *Can J Psychol* **41** (1987), 365–378.

[17] D.E. Meyer, R.A. Abrams, S. Kornblum, C.E. Wright and J.E.K. Smith, Optimality in human motor performance: ideal control of rapid aimed movements, *Psychol Rev* **95** (1988), 340–370.

[18] D.E. Meyer, J.E.K. Smith, S. Kornblum, R.A. Abrams and C.E. Wright, Speed-accuracy tradeoffs in aimed movements: Toward a theory of rapid voluntary action, in: *Attention and Performance XIII*, M. Jeannerod, ed., Lawrence Erlbaum Associates, Inc.: Hillsdale, 1990, pp. 173–226.

[19] D.E. Meyer, J.E.K. Smith and C.E. Wright, Models for the speed and accuracy of aimed movements, *Psychol Rev* **89** (1982), 449–482.

[20] J. Paillard, Apraxia and the neurophysiology of motor control, *Phil Trans R Soc Lond B Biol Sci* **298** (1982), 111–134.

[21] J. Paillard, Introductory lecture: The functional labelling of neural codes, in: *Neural Coding of Motor Performance, Vol. 7, Exp Brain Res Suppl*, J. Massion, J. Paillard, W. Schultz and M. Wiesendanger, eds, Springer-Verlag: Berlin, 1983, pp. 1–19.

[22] H.L. Paulson and M.B. Stern, Clinical manifestations of Parkinson's disease, in: *Movement disorders: Neurologic principles and practice*, R.L. Watts and W.C. Koller, eds, MacGraw-Hill: New York, 1997, pp. 183–199.

[23] M.K. Rand, J.L. Alberts, G.E. Stelmach and J.R. Bloedel, The influence of movement segment difficulty on movements with two-stroke sequence, *Exp Brain Res* **115** (1997), 137–146.

[24] A.F. Sanders, Structural and functional aspects of the reaction process, in: *Attention and Performance VI*, S. Dornic, ed., Lawrence Erlbaum Associates: Hillsdale, 1977, pp. 3–25.

[25] R.A. Schmidt, A schema theory of discrete motor skill learning, *Psychol Rev* **82** (1975), 225–260.

[26] R.A. Schmidt, Control processes in motor skills, *Exerci Sport Sci Rev* **4** (1976), 229–261.

[27] R.A. Schmidt and T.D. Lee, *Motor control and learning: A behavioral emphasis*, Champaign: Human Kinetics Publishers: Champaign, 1999.

[28] R.A. Schmidt, H.N. Zelaznik and J.S. Frank, Sources of inaccuracy in rapid movement, in: *Information processing in motor control and learning*, G.E. Stelmach, ed., Academic Press: New York, 1978, pp. 183–203.

[29] R.A. Schmidt, H.N. Zelaznik, B. Hawkins, J.S. Frank and J.T. Quinn, Jr., Motor-output variability: a theory for the accuracy of rapid motor acts, *Psychol Rev* **86** (1979), 415–451.

[30] M.R. Sheridan, K.A. Flowers and J. Hurrell, Programming and execution of movement in Parkinson's disease, *Brain* **110** (1987), 1247–1271.

[31] B. Sidaway, H. Sekiya and M. Fairweather, Movement variability as a function of accuracy demand in programmed serial aiming responses, *J Mot Behav* **27** (1995), 67–76.

[32] J.F. Soechting, Effect of target size on spatial and temporal characteristics of a pointing movement in man, *Exp Brain Res* **54** (1984), 121–132.

[33] S. Sternberg, The discovery of processing stages: extensions of Donders' method, *Acta Psychol* (*Amst*) **30** (1969), 276–315.

[34] J. Theios, The components of response latency in simple human information processing tasks, in: *Attention and Performance V*, P.M.A. Rabbit and S. Dornic, eds, Academic Press: London, 1975, pp. 418–440.

[35] D.T. Wade, Measuring arm impairment and disability after stroke, *Int Disabil Stud* **11** (1989), 89–92.

[36] N. Walker, D.A. Philbin and A.D. Fisk, Age-related differences in movement control: adjusting submovement structure to optimize performance. *J Gerontol B Psychol Sci Soc Sci* **52** (1997), 40–52.

[37] P. Waters and P.L. Strick, Influence of 'strategy' on muscle activity during ballistic movements, *Brain Res* **207** (1981), 189–194.

[38] K.M. Wood, J.D. Edwards, O.J. Clay, V.G. Wadley, D.L. Roenker and K.K. Ball, Sensory and cognitive factors influencing functional ability in older adults, *Gerontology* **51** (2005), 131–141.
